# Targeting of Rho Kinase Ameliorates Impairment of Diabetic Endothelial Function in Intrarenal Artery

**DOI:** 10.3390/ijms141020282

**Published:** 2013-10-14

**Authors:** Hongping Yin, Hailong Ru, Liping Yu, Yanhua Kang, Guohua Lin, Chuanfei Liu, Lixian Sun, Liyun Shi, Qinghua Sun, Cuiqing Liu

**Affiliations:** 1 Laboratory Center for Medical Science, Hangzhou Normal University, Hangzhou 310036, China; E-Mail: yhp-1975@163.com; 2 College of Medicine, Hangzhou Normal University, Hangzhou 310036, China; E-Mails: rhlhz@163.com (H.R.); 13758140215@163.com (Y.K.); linguohuahznu@gmail.com (G.L.); chuanfeiliu@sohu.com (C.L.); shi_liyun@msn.com (L.S.); 3 Experimental Animal Center, Hangzhou Normal University, Hangzhou 310036, China; E-Mail: luyuxuanhn@aliyun.com; 4 Department of Cardiology, Chengde Medical College, Chengde 067000, China; E-Mail: lixiansun01@126.com; 5 College of Public Health, the Ohio State University, Columbus, OH 43210, USA; E-Mail: qinghua.sun@osumc.edu

**Keywords:** Rho kinase, endothelial dysfunction, diabetic nephropathy, oxidative stress

## Abstract

Endothelial dysfunction in kidney vasculature is the initial and key element for nephropathy in diabetes mellitus. Accumulating evidence suggests the protective role of Rho kinase inhibitors in endothelial dysfunction via modulating eNOS activity and NO production. However, the role of Rho kinase in diabetes-related endothelial dysfunction in kidney vasculature and the relevant mechanisms remain unknown. We assessed whether pharmacological inhibition of Rho kinase attenuates endothelial dysfunction in intrarenal arteries from type 1 diabetic rats. Fasudil, a Rho kinase inhibitor effectively decreased the phosphorylated level of MYPT1 without affecting the expression of ROCKs in the kidney. Fasudil treatment showed no improvement in diabetes-related abnormality in metabolic indices, but it significantly ameliorated endothelial dysfunction in intrarenal arteries and lessened the mesangial matrix expansion in the kidney cortex. Mechanistically, superoxide production in the intrarenal artery and NOX4 member of NADPH oxidase in the renal cortex that contribute to diabetic nephropathy were also prevented by the Rho kinase inhibitor. In conclusion, the present results indicate that Rho kinase is involved in endothelial dysfunction in type 1 diabetes via enhancement of oxidative stress and provides new evidence for Rho kinase inhibitors as potential therapeutic agents for the treatment of diabetic nephropathy.

## 1. Introduction

Rho GTPase and its downstream effector Rho-kinase (Rho-associated, coiled-coil-containing protein kinase, ROCK) play important roles in the pathogenesis of systemic vascular diseases [1] by sensitizing the contractile machinery to Ca^2+^[2,3], and negatively regulating NO production [4,5] in the vascular system. Recently, the Rho/Rho-kinase pathway has been implicated in diabetes, including erectile dysfunction, cardiomyopathy, retinopathy, cerebro-vascular disease and nephropathy [6–10]. Thus, Rho kinase inhibitors may represent another group of therapeutic agents for diabetic complications [11].

Diabetic nephropathy is the leading cause of end-stage renal disease. Diabetes mellitus is characterized by systemic endothelial dysfunction [12], which is the initial and key element for the development of nephropathy. Studies have demonstrated that treatment with fasudil, a Rho-kinase inhibitor, prevents the development of diabetic nephropathy in streptozotocin-treated rats [13] or insulin-resistant diabetic rats [14]. In addition, Rho kinase may play an important role in renal fibrosis by enhancing signaling pathways involving transforming growth factor-beta, angiotensin II and nuclear factor-κB [15,16]. However, the underlying mechanisms are far from clear. A growing body of evidence also suggests the protective role of Rho kinase inhibitors in diabetes-induced vascular (aorta) dysfunction which can be due to inhibition of eNOS activity thereby less endothelial NO-production [17], but its role in the endothelial function in the kidney vasculature and the relevant mechanisms remain unknown. In the present study, therefore, we determined the contribution of Rho kinase to intrarenal artery endothelial dysfunction using an STZ-induced diabetes model and investigated the possible mechanisms.

## 2. Results and Discussion

### 2.1. Chronic Rho Kinase Inhibition Does Not Meliorate the Metabolic Parameters in Diabetic Rats

**Figure 1:**
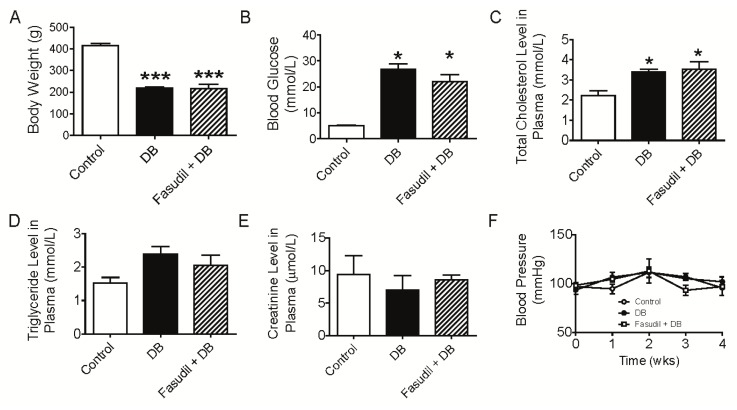

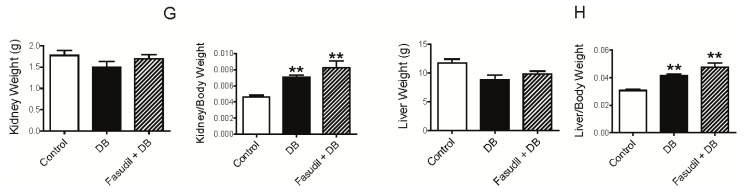
Effects of Rho kinase inhibition on metabolic parameters in diabetic rats. (**A**) Body weight of rats after 4-week treatment with fasudil; (**B**) Blood glucose of rats after 4-week treatment with fasudil; (**C**–**E**) Total cholesterol level (**C**), triglyceride level (**D**) and creatinine level (**E**) in plasma of rats after 4-week treatment with fasudil; (**F**) Blood pressure of rats during 4-week treatment with fasudil; (**G**,**H**) kidney weight, kidney weight/body weight ratio (**G**) and liver weight, liver weight/body weight ratio (**H**) of rats after 4-week treatment with fasudil. *p < 0.05, **p < 0.01, ***p < 0.001 compared with control group. Data are mean ± S.E.M. from five to six different rats.

There were no differences in body weight or blood glucose between the groups at baseline (data not shown). Diabetic rats demonstrated reduced weight gain and hyperglycemia (Figure 1A,B), indicating a successful induction of diabetic model. Total cholesterol was significantly increased following STZ injection while there were no differences in plasma triglyceride or creatinine between the two groups, although there was a trend towards increment in tryglyceride in STZ-treated rats (Figure 1C–E). There is a vast literature showing an increase in relative kidney size in diabetic nephropathy in humans and rodent diabetic models. Consistent with it, STZ injection resulted in renal hypertrophy (as assessed by kidney weight/body weight) at the end of the treatment period, although no difference was observed by comparing organ weight itself between groups (Figure 1G). Similar results were noticed with liver (Figure 1H). However, treatment with Rho kinase inhibitor, fasudil, had no effects on these general characteristics (Figure 1), which was in agreement with the study by Komers *et al.* [18].

In contrast to the decreases in blood pressure following Rho kinase inhibition (by structurally different Rho kinase inhibitors, fasudil and Y27632) in a STZ-induced diabetic model [18], fasudil treatment failed to reduce blood pressure during the 4-week treatment period in the present study. As shown in Figure 1F, although fasudil treatment induced a trend to decrease blood pressure after 1-week and 3-week of treatment, this small decrease did not reach a significant difference. The discrepancy in effect on blood pressure in response to fasudil treatment was likely due to different sensitivity of methodology to measure blood pressure with direct catheterizing into femoral artery in Komers’ study and indirect tail-cuff manometry in the present study. Another possibility could be that blood pressure was measured instantly after one single dose infusion of Rho kinase inhibitors (fasudil or Y27632) in Komers’ study but at fixed time consecutively during the whole period with daily treatment of fasudil in our project [18]. This rationale was supported by the study with a long-term fasudil treatment experimental contex [19]. Considering the characteristic of autoregulation in blood pressure, the compensatory upregulation of blood pressure after fasudil administration should not be excluded, which may mask the acute depressor response to fasudil treatment.

### 2.2. Chronic Rho Kinase Inhibition Improves Endothelial Function in Intrarenal Artery from Diabetic Rats

Diabetes, which is more than a problem of hyperglycemia, is also characterized by several functional and structural alterations in the vasculature. A great amount of studies have demonstrated the occurrence of endothelial dysfunction in the course of diabetic vascular complications [17,20]. Since endothelium function is evidenced not only by endothelium-dependent relaxation but also endothelium-dependent contraction, the effect of blockade of Rho kinase on both of them were examined in the intrarenal artery from diabetic rats.

#### 2.2.1. Effect of Rho Kinase Inhibition on Endothelium-Dependent Relaxation in Intrarenal Artery

**Figure 2:**
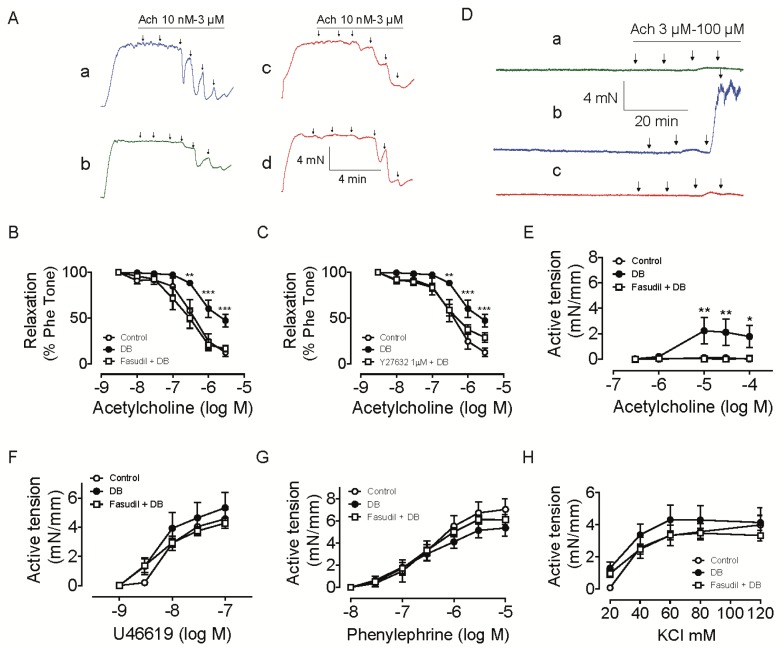
Effect of Rho kinase inhibition on vascular dysfunction in intrarenal artery from diabetic rats. (**A**) Representative traces showing acetylcholine (ACh)-induced relaxations in phenylephrine (Phe)-contracted artery rings from control rats (**a**); diabetic rats (**b**); diabetic rats treated with fasudil (**c**) and diabetic rats but with rings acutely incubated in 1 μM Y27632 for 30 min (**d**); (**B**) Concentration-response curves for ACh in artery rings from rats treated with or without fasudil; (**C**) Concentration-response curves for ACh in artery rings with or without acute incubation of Y27632; (**D**) Representative traces showing acetylcholine (ACh)-induced contractions of artery rings in presence of L-NAME from control rats (**a**); diabetic rats (**b**) and diabetic rats treated with fasudil (**c**); (**E**) Concentration-response curves in artery rings for ACh with L-NAME pretreatment; (**F**–**H**) Concentration-response curves in artery rings in response to U46619 (**F**), phenylephrine (**G**) and KCl (**H**). *p < 0.05, **p < 0.01, ***p < 0.001 compared with control group at respective concentration. Data are mean ± S.E.M. from four–five different animals.

Traces in Figure 2A show that acetylcholine caused a concentration-dependent relaxation of intrarenal arteries pre-constricted with phenylephrine. The endothelium-dependent vasodilatation in the intrarenal arteries was attenuated in diabetic group (the maximum relaxation: 87.4 ± 4.6% in control group, 52.6 ± 7.0% in diabetic group; pD_2_: 6.4 ± 0.13 in control group, 6.2 ± 0.10 in diabetic group) indicating endothelial dysfunction in kidney vasculature (Figure 2A(a,b),2B), which may play a significant role in the pathogenesis of nephropathy in diabetic animals. Fasudil treatment resulted in an improvement in the attenuated acetylcholine-induced relaxation (the maximum relaxation: 52.6 ± 7.0% in diabetic group, 83.2 ± 3.4% in fasudil + diabetic group; pD_2_: 6.2 ± 0.10 in diabetic group and 6.7 ± 0.13 in fasudil + diabetic group) (Figure 2A(b,c),2B). Similarly, acute treatment of arteries from diabetic rats with Y27632 at 1 μM improved the endothelial function, with maximum relaxation of 71.1 ± 5.2% and pD_2_ of 6.5 ± 0.11 (Figure 2A(d),2C). These data suggest a possible role of Rho kinase activity in the attenuated endothelial function in diabetic rats. Although the impaired NO sensitivity of vascular smooth muscle cells can also contribute to endothelial dysfunction, our previously published data have demonstrated the detrimental effect of Rho kinase activation on the function of both endothelium and vascular smooth muscle cells [21,22]. Thus, it is reasonable to conclude that Rho kinase is involved in endothelial dysfunction in the current diabetic model. Early stages of nephropathy in diabetes are characterized by specific haemodynamic changes characterized by elevations in glomerular filtration rate or increases in filtration fraction. It has been demonstrated previously that Rho kinase inhibitors administration led to increases in effective renal plasma flow, reductions in renal vascular resistance and decreases in filtration fraction in diabetic animals [18]. Our results may provide direct evidence for the renal haemodynamic response to Rho kinase inhibitors, indicating a contribution of Rho A/Rho kinase pathway to haemodynamic changes in the diabetic kidney and nephropathy.

#### 2.2.2. Effect of Rho Kinase Inhibition on Endothelium-Dependent Contraction in Intrarenal Artery

In the presence of NG-nitro-l-arginine methyl ester (L-NAME), acetylcholine elicited pronounced contraction in arteries from diabetes rats, with a maximal response of ~2.2 ± 1.03 mN/mm segment (Figure 2D(a,b),2E). The acetylcholine-induced contraction was inhibited in rings from rats treated with fasudil (0.06 ± 0.05 mN/mm segment), in parallel with the curve from control group (0.12 ± 0.06 mN/mm segment), *p* < 0.05 compared with diabetes group (Figure 2D(a,b),2E). Rho kinase inhibition did not modulate contractions induced by phenylephrine, U46619 or KCl in the current experimental condition (Figure 2F–H).

**Figure 3:**
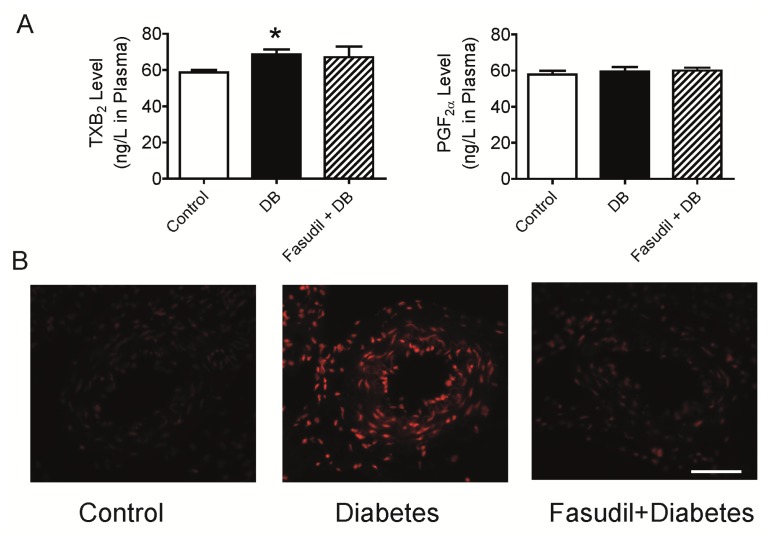

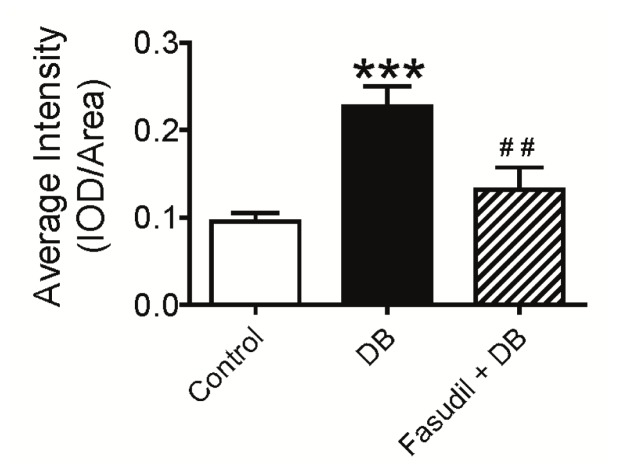
Effects of Rho kinase inhibition on oxidative stress in intrarenal artery and EDCFs in plasma from diabetic rats. (**A**) The concentrations of TxB_2_ and PGF_2α_ in plasma of rats after 4-week treatment with fasudil. (**B**) Fluorescent photomicrographs at identical settings of sections of intrarenal artery labeled with dihydroethidium and quantification of the fluorescent ethidium signal in the artery by average intensity. Scale bar is 50 μm shown in the picture. *p < 0.05, ***p < 0.001 compared with control group; ^##^*p* < 0.01 when compared diabetic rats with and without treatment of fasudil. Data are mean ± S.E.M. from four–six different animals.

It is well known that endothelium-dependent contractions generally involve the generation of COX1- and/or COX2-derived products and the activation of smooth muscle thromboxane prostanoid receptor (TP receptor). Depending on the model, TxA_2_, PGH_2_, PGF_2α_, PGE_2_, and paradoxically PGI_2_ can all act as endothelium-derived contracting factors (EDCFs) [23–25]. Thus we measured some of the ECDFs in the plasma. As shown in Figure 3A, STZ injection resulted in an increase in plasma TxB_2_ level in plasma but no alteration of plasma PGF_2α_ level was observed. Treatment of the diabetic rats with Rho kinase inhibitor, fasudil, did not affect the plasma content of TxB_2_ or PGF_2α_ (Figure 3A). TxB_2_ is the derivative of TxA_2_, an unstable prostanoid metabolite of arachidonic acid, which activates the TP receptor and leads to activation of Rho A-Rho kinase pathway. Thus, the increase of TxB_2_ level in plasma helps to explain the increased acetylcholine-induced endothelium-dependent contraction in the intrarenal artery of diabetic rats. Since Rho kinase is the downstream signals of EDCFs, it is no surprising to observe that fasudil treatment did not decrease the production of TxB_2_. Similarly, the acetylcholine-induced contraction was almost abolished by fasudil treatment. Hence, fasudil contributed to the attenuated endothelium-dependent vasocontraction, which may due to inhibition of the Rho kinase activated by TxA_2_, but not related to synthesis of these endothelium-derived contracting factors.

Since endothelium controls the tone of the underlying vascular smooth muscle cells through releasing NO during both physiological and pathological conditions, many studies have demonstrated that hyperglycemia impairs vascular function via inhibition of endothelium-dependent vasodilatation, accompanied by selective impairment of NO-dependent component of vasodilatation [26]. Although we did not detect the eNOS expression, eNOS activity, or NO production directly in the isolated artery due to limited availability of renal arteries, our previous study showed that eNOS activity was inhibited by Rho kinase activation, leading to decrease in NO production and endothelial dysfunction in both the cellular and tissue levels [21]. Consistent with it, an involvement of Rho kinase pathway in the mechanical activity of arteries via decrease in both eNOS expression and NO production in endothelium were reported in arteries from diabetic animals [27,28]. As stated previously, TxA_2_ is the key activator for TP receptor which results in Rho kinase activation. Considering TxB_2_ (the derivative of TxA_2_) was increased in plasma of diabetic rats, therefore, it is postulated that hyperglycemia may stimulate production of TxA_2_, leading to endothelial dysfunction through TP receptor-Rho kinase pathway.

#### 2.2.3. Effect of Rho Kinase Inhibition on O_2_^•−^ Production in the Intrarenal Artery

Except for the beneficial effects on endothelium-dependent relaxation, endothelial lining, and serum nitrite/nitrate concentration, inhibition of Rho kinase by fasudil has also been demonstrated to prevent diabetes-induced increase in serum thiobarbituric acid reactive substances (TBARS) in diabetic rats [29]. Y27632, another Rho kinase inhibitor, was reported to effectively inhibit nicotinamide adenine dinucleotide phosphate (NADPH) oxidase p22phox, p47phox, gp91phox and LOX-1 expression, up-regulate eNOS expression in the hypertrophy ventricular from Dahl salt-sensitive hypertensive rats [30]. These data led us to investigate the level of oxidative stress in the intrarenal artery. As expected, Figure 3B shows result of DHE staining for superoxide measurement. Quantification of the fluorescent signal showed a ~2.1-fold increase in O_2_^•−^ production in the intrarenal artery from diabetic rats, compared to control (Figure 3B, *p* < 0.05 *vs.* control). Fasudil treatment resulted in noticeable decrease in superoxide content in this area (Figure 3B), *p* < 0.05 *vs.* diabetic group. These results support the hypothesis that inhibition of Rho kinase and consequent reversal of oxidative stress may contribute to the attenuated endothelial dysfunction in renal arteries.

### 2.3. Chronic Rho Kinase Inhibition Ameliorates Development of Diabetic Nephropathy in Renal Cortex from Diabetic Rats

#### 2.3.1. Effect of Rho Kinase Inhibition on Mesangial Expansion and Glomerular hypertrophy

**Figure 4:**
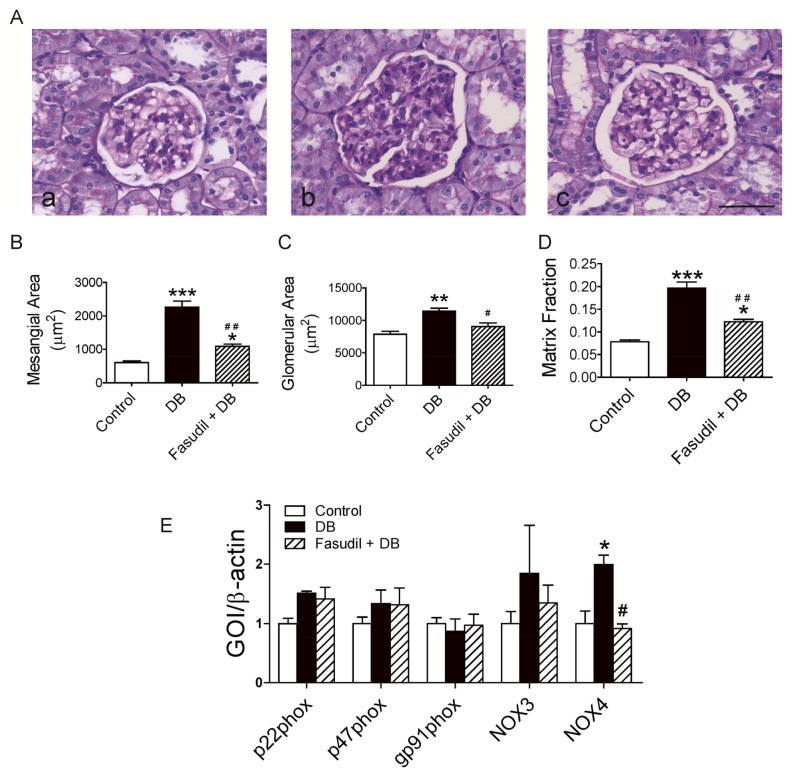
Effects of Rho kinase inhibition on mesangial matrix expansion and expression of NADPH oxidase subunits in renal cortex from diabetic rats. (**A**) Representative photomicrographs of PAS–stained kidney. a, control rats; b, diabetic rats; c, diabetic rats treated with fasudil. Scale bar is 50 μm shown in the picture. B–D. Quantitative analysis of mesangial area (**B**), glomerular area (**C**), and matrix fraction (**D**); (**E**) Effect of Rho kinase inhibition on gene expression of NADPH oxidase subunits/members in the renal cortex of diabetic rats. *p < 0.05, **p < 0.01, ***p < 0.001 compared with control group; ^#^*p* < 0.05, ^##^*p* < 0.01 when compared diabetic rats with and without treatment of fasudil. Data are mean ± S.E.M. from five different animals.

Mesangial expansion and glomerular hypertrophy are the most striking morphologic characteristics of diabetic nephropathy. As shown in Figure 4A, accelerated mesangial expansion, characterized by an increase in PAS-positive mesangial matrix area, was shown in diabetic rats compared with control rats (Figure 4A(a,b), 4B). This alteration in glomerular structure was noticeably ameliorated in fasudil-treated diabetic rats (Figure 4A(c), 4B). Glomerular hypertrophy was evidenced by glomerular area (defined by tracing along the outline of the capillary loop). Similarly, the glomerular area was increased in diabetic rats and was reduced by fasudil treatment (Figure 4A,C). Accordingly, the matrix fraction calculated by the ratio of the mesangial area to the glomerular area increased in diabetic rats compared with control rats, and fasudil significantly ameliorated the increase in matrix fraction (Figure 4D). Consistent with studies from other groups [19,31], these histological analysis indicated that suppression of Rho kinase with fasudil ameliorated these injuries in diabetic rats and supported the important role of Rho/Rho kinase as a significant contributor to the development of diabetic glomerulosclerosis. Since it was still in the earlier stages of nephropathy for the current experiment, we only observed a lightly widened tubular lumen and fine vacuoles in the cortex area of diabetic rats with no improvement in response to fasudil treatment (data not shown), while Masson’ trichrome staining showed little collagen deposition in the cortex area in all groups (data not shown).

#### 2.3.2. Effect of Rho Kinase Inhibition on NOX4 Expression in Renal Cortex

It has been proposed that Rho kinase activation is dependent on reactive oxygen species production and down-regulation of the expression of NADPH oxidase (the major source of oxidative stress) [13]. This finding led us to investigate whether oxidative stress is involved in the attenuation of glomerulosclerosis by Rho kinase inhibition. To assess the effect of Rho kinase inhibition on NADPH oxidase gene expression, we evaluated expression of genes encoding NADPH oxidase, including NOX3, NOX4, p22phox, p47phox and gp91phox in the renal cortex tissue from rats treated with and without fasudil. As shown in Figure 4E, there was no difference in NOX3, p22phox, p47phox or gp91phox between groups. However, we observed a ~2-fold increase in NOX4 expression in cortex from diabetic rats, which is totally inhibited by fasudil treatment (Figure 4E). In line with Gojo’s study [13], the expression analysis of subunits or members of NADPH oxidase in the renal cortex demonstrated that the normalization of up-regulated NOX4, but not other subunits/members may contribute to the amelioration in nephropathy development in diabetic rats treated with fasudil. Although NOX4 has been reported to constitutively generate hydrogen peroxide, Lee *et al.* clearly demonstrated that NOX4 is necessary for Ang II-induced intracellular O_2_^•−^ production since the increased NADPH oxidase activity and intracellular O_2_^•−^ caused by AngII was abolished by down-regulation with specific siRNA of the NOX4 [32]. It can be assumed that the NOX4-mediated reactive oxygen species, which is reduced by Rho kinase inhibition, may contribute to the pathologic alteration in the kidney cortex. The molecular mechanism by which Rho kinase inhibition reduces the progression of diabetic nephropathy and whether NOX4 regulates the endothelial function in intrarenal artery await further investigation.

### 2.4. Chronic Rho Kinase Inhibition Alters No Rho-Kinase Expression but Decreased Rho Kinase Activity in the Kidney

**Figure 5:**
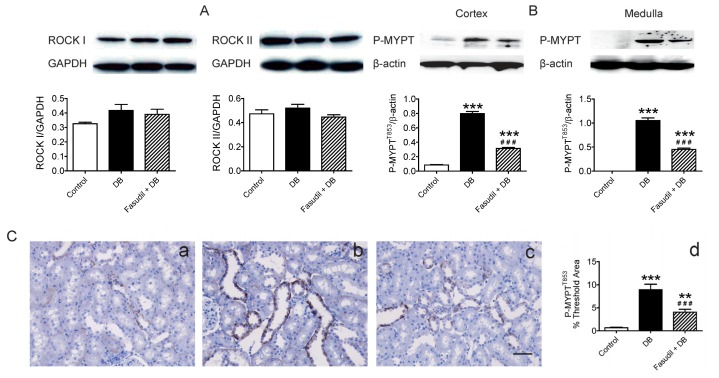
Effects of Rho kinase inhibition on Rho kinase expression and activity in kidney of diabetic rats. (**A**) Representative bands and analysis of protein levels of ROCK I and ROCK II in renal cortex of rats; (**B**) Representative bands and analysis of protein levels of phosphorylated form of MYPT1 (P-MYPT1) from the renal cortex and medulla; (**C**) Quantitative analysis and representative images of immunohistochemistry for P-MYPT1 from the renal cortex. a, control rats; b, diabetic rats; c, diabetic rats treated with fasudil; d, statistical analysis of immunohistochemistry for P-MYPT1 in renal cortex. **p < 0.01, ***p < 0.001 compared with control group; ^###^*p* < 0.001 when compared diabetic rats with and without treatment of fasudil. Data are mean ± S.E.M. from five different rats.

As described by Nunes *et al.*, up-regulation of RhoA/Rho-kinase pathway is a key link among many diseases including diabetes [33,34]. Thus, we further investigated the impact of fasudil on Rho kinase at the molecular level. There are two isoforms of Rho kinase, ROCK 1 and ROCK 2. Cicek *et al.* measured 2.5-fold higher ROCK2 protein level in endothelium-intact aortic rings from diabetic rats compared to those of the control while its level in endothelium-denuded aortic rings was similar among these two groups [17]. Due to the limit amount of intrarenal artery, it is difficult to test the ROCK protein in the vasculature itself in the present study. However, we did examine the expression of the two isoforms of Rho kinase (ROCK 1 and ROCK 2) in the renal cortex and found no difference in the expression of either isoform (Figure 5A).

Rho A/Rho kinase acts as mediators of agonist-induced vascular smooth muscle contraction as well as in the control of myogenic tone of resistance arteries. Activation of Rho kinase can induce phosphorylation of myosin phosphatase target subunit 1 (MYPT1), a subunit of MLCP, which in turn inhibits MLCP activity and thus leading to vascular contraction [2,3]. To confirm the effect of fasudil as a Rho kinase inhibitor, we examined Rho kinase activity characterized by the phosphorylated form (T853) of MYPT1 in the kidney cortex and medulla. As shown in Figure 5B, the levels of phosphorylated MYPT1 in both area were increased in non-treated diabetic rats as compared with control animals with no STZ injection (Figure 5B), and this increase was significantly inhibited by fasudil treatment. Consistent with it, immunohistochemistry staining showed the phosphorylation of MYPT1, mainly located in the tubular epithelial cells, was enhanced in diabetic kidney and partially blocked by Rho kinase inhibition (Figure 5C). These results provided direct support for the ROCK-dependent effect of fasudil in the present study and suggested that the beneficial effect of fasudil is likely mediated through reversing the enhanced Rho kinase activity in diabetic rats.

In conclusion, Rho kinase inhibition with fasudil initiated at the onset of diabetes had blood pressure- and blood glucose-independent beneficial effects on the endothelial function of intrarenal artery in STZ-induced diabetic rats, associated with a modest reduction in superoxide production in the artery area but with no decrement in plasma levels of EDCFs. These beneficial functional effects were accompanied with beneficial structural effects evidenced by inhibition of mesangial expansion and glomerular hypertrophy, associated with reduced expression of NOX4, a NADPH oxidase member in the kidney cortex.

However, the present study has important limitations that must be acknowledged. Firstly, eNOS-NO signaling pathway plays a pivotal role in endothelial dysfunction, while neither eNOS activity/expression nor NO production in the artery was examined in the present study due to the limited amount of tissue (isolated intrarenal arteries). In addition, we could not examine the protein and/or mRNA expression of Rho kinase, and NAD(P)H oxidase subunits or members in the examined artery itself. We have thus no evidence to support the effect of fasudil on these processes as being directly responsible for the salutary effects. Notwithstanding these limitations, our results support the approaches to target Rho kinase in diabetic nephropathy.

## 3. Experimental Section

### 3.1. Animal Model

Male Sprague-Dawley rats (180–200 g) were housed in a certified animal care facility with free access to food and water. Diabetes was induced by intraperitoneally injecting streptozotocin (55 mg/kg in citrate buffer (pH 4.5) [29,35]. Control rats were injected with vehicle alone. Diabetes was verified 72 h later by evaluating blood glucose levels with blood-glucometer (Life Scan, Milpitas, CA, USA). Rats having blood glucose level of 16.7 mM or greater were considered to be diabetic.

Three days after the verification of diabetes diabetic rats were divided into two groups: an untreated group and a group treated with fasudil (Rho kinase inhibitor, 10 mg/kg/day). The treatment lasted 4 weeks. The dosage of fasudil used in this study was based on the study by Tsounapi *et al.* [36]. Animal experiments were in accordance with the National Institute of Health Guide for the Care and Use of Laboratory Animals, with the approval of the Animal Care and Use Committee at Hangzhou Normal University.

### 3.2. Measurement of Blood Pressure and Plasma Analysis

During the experiment, mean blood pressure and pulse were measured weekly in conscious rats using a computerized non-invasive tail-cuff manometry system (Soften BP-98A, Tokyo, Japan). The parameters were determined as the average of measurement over 3 consecutive days. In addition, during each measurement day, 10 acclimatization cycles were followed by 20 measurement cycles, which were collected to obtain the average values for blood pressure and pulse for each rat. At the end of the experiment, rats were fasted overnight and blood glucose was measured.

Total cholesterol concentration, triglycerides and creatinine levels in plasma were determined using commercial kits (Diasys Diagnostic Systems, Shanghai, China) with automatic biochemical analyzer (Hitachi 7020, Tokyo, Japan).

TxB_2_, PGF_2α_ in plasma were determined using ELISA kits (R&D Systems China Co. Ltd., Shanghai, China) according to manufacturer’s instructions.

### 3.3. Vascular Reactivity

At the end of the experiment, rats were killed by cervical dislocation. Vasoreactivity of renal arteries were studied as described [37]. Briefly, after the abdominal cavity was opened, the kidneys were removed and placed in ice-cold Krebs solution (in mM): 119 NaCl, 4.7 KCl, 2.5 CaCl_2_, 1 MgCl_2_, 25 NaHCO_3_, 1.2 KH_2_PO_4_, and 11 d-glucose. The intralobar renal arteries (mean external diameter of ~350 μm) were dissected from both kidneys, and each artery was cleaned of adhering tissues and cut into ring segments of ~2 mm in length. Each segment was mounted in a Multi Myograph System (Danish Myo Technology, Aarhus, Denmark), with two tungsten wires (each 40 μm in diameter) being inserted through the segment’s lumen, and each wire was fixed to the jaws of a myograph. The organ chamber was filled with 5 ml Krebs solution and oxygenated with a 95% O_2_–5% CO_2_ gas mixture. Krebs solution in the chamber was maintained at 37 °C using a built-in heat-exchanger device to give a pH value of ~7.4. The basal tension was regulated to 2 mN, an optimal tension. After stabilizing for 90 min, the vascular contraction, endothelium-derived relaxations (EDRs) and endothelium-derived contractions (EDCs) were recorded. Vascular contraction was induced by various constrictors including phenylephrine (30 nM–10 μM), U46619 (3 nM–100 nM) and KCl (20 mM–120 mM). The EDRs were induced by cumulative addition (10 nM–3 μM) of acetylcholine in arteries that had been precontracted by phenylephrine (1 μM), and the EDCs were elicited by acetylcholine (1 μM–100 μM) after pretreatment with 100 μM L-NAME, which inhibited the production of endothelium-derived NO. To further evaluate the involvement of Rho kinase, arteries from diabetic rats were first acutely exposed for 30 min to Y27632 (another selective inhibitor of Rho kinase at 1 μM), and then contracted with phenylephrine prior to the addition of acetylcholine.

### 3.4. Immunoblotting

Protein levels were determined by Western blotting method. Kidney cortex tissue was homogenized in M-PER Mammalian protein extraction reagent (Thermo Scientific, Rockford, IL, USA) on ice. Equal quantities of protein for respective tissue were separated by 10% SDS-PAGE. Following transfer to immobilon-P polyvinylidene difluoride (PVDF) membrane and blocking with 5% nonfat milk, the blot was incubated with different primary antibodies: ROCK I, ROCK II (1:500, 1:1000 respectively, BD Bioscience, San Jose, CA, USA). The immunoblots were incubated with a secondary goat anti-mouse antibody conjugated with horseradish peroxidase (Santa Cruz, INC, Santa Cruz, CA, USA) and visualized with enhanced chemiluminescence, and the autoradiograph was quantitated by densitometric analysis with ImageJ software (http://www.onlinedown.net/soft/87295.htm (accessed on 12 February 2013)). GAPDH was used as control reference.

### 3.5. Histology and Immunohistochemistry

The paraffin-embedded kidney specimens were cut into slices of 4 μm in thickness and mounted on glass slides. Thin sections were routinely deparaffinized and rehydrated in distilled water. Some sections were stained with hematoxylineosin (H&E) to observe the tissue morphology. Some sections were performed with Periodic Acid Schiff (PAS) staining. Briefly, deparaffinized sections were oxidized in 0.5% periodic acid solution for 5 minutes and then stained in Schiff reagent for 15 min. After washing in lukewarm tap water for 5 min, then were dehydrated in a graded series of ethanol. Finally, cover slips were placed on glass slides with a mounting medium, neutral balsam and observed under a light microscope. Some other deparaffinized sections were stained with immunohistochemistry to test P-MYPT1 with EnVision+ ^™^Peroxidaxe kit (DAKO). Briefly, deparaffinized sections were subjected to heat-induced antigen retrieval by incubation in citrate buffer solution (pH = 6) for 20 min at 95 °C, cooled down to room temperature, and washed with phosphate buffered saline (PBS). These sections were then treated with 3% hydrogen peroxide at room temperature for 10 minutes in order to inhibit endogenous peroxidase activity. After rinsing, the sections were blocked with 1% BSA in PBS and incubated with rabbit anti-rat P-MYPT1 (myosin phosphatase target subunit 1, phosphor T853 at 1:100, abcam) at 4 °C overnight. The sections were incubated within EnVision solution at 37 °C for 30 min, and then developed by DAB according to the manufacturer’s instruction (Sigma). Counter-staining on nucleus was performed with haematoxylin for 1 min and then blued with tap water. After dehydration, the sections were mounted with Permount^®^. Negative control was performed in the absence of primary antibody. Sections were acquired with Olympus BX51 DP70 microscope (Olympus Tokyo, Japan). Automatic computer-based analysis was performed using Image-Pro Plus 7.0. Data were expressed as average intensity of threshold area.

### 3.6. Quantitative RT-PCR

RT-PCR was performed using RNA extracted from kidney cortex tissue of the experimental rats. Total RNA was isolated with Trizol (Invitrogen, Carlsbad, CA, USA) according to the manufacturer’s protocol. cDNA was reversely transcribed using High Capacity cDNA Transcription kit (Applied Biosystems, Carlsbad, CA, USA). Quantitative polymerase chain reaction (qPCR) was performed in duplicate using the ABI 7300. “No template,” cDNA negative controls were included for each gene set in all PCR reactions to detect contamination. The thermocycler program was set as 10 min at 95 °C, followed by 50 cycles of at 95 °C for 15 s and 60 °C for 1 min. The expression level for each gene was calculated using the Δ*C*t method relative to β-actin. The sequences of all primers except for those used in hypothalamus tissue are listed in Table 1.

### 3.7. Localization and Quantification of Superoxide Anion by Dihydroethidium

The oxidative fluorescent probe dihydroethidium (DHE) was used to evaluate *in situ* O_2_^•−^ production on histological sinus sections of 10-μm thickness as previously described [38]. DHE is a cell permeable dye that is oxidized by O_2_^•−^ to 2-hydroxyethidium, which subsequently intercalates with DNA and is trapped within cell nuclei. DHE 10 μM (Molecular Probes, Biyuntian, Haimen, China) was topically applied to each tissue section. Slides were incubated in a light-protected humidified chamber at room temperature for 30 minutes, rinsed with PBS, and analyzed with a fluorescent Olympus IX51 + X-CITE SERIES 120Q (Olympus, Tokyo, Japan). Acquisition settings of the camera were identical for images of different specimens. Automatic computer-based analysis was performed using Image-Pro Plus 7.0. Data were expressed as average intensity of threshold area.

### 3.8. Chemicals

9,11-dideoxy-11α,9α-epoxy-methanoprostaglandin F2α (U46619), phenylephrine, acetylcholine, NG-nitro-L-arginine methyl ester (L-NAME) were purchased from Sigma. Y27632 was a kind gift from Dr. Yu Huang from Chinese University of Hong Kong. Fasudil was purchased from Chase Sun, China. U46619 was dissolved in dimethyl sulfoxide (DMSO) as stock solution and all other drugs in double-distilled water. Stock solutions were stored at −20 °C. Desired dilution was prepared in Krebs solution shortly before experimentation.

### 3.9. Data Analysis

Data are means ± standard error of the mean for the number of animals. Graphpad Prism software (Version 5, GraphPad Software, Inc., San Diego, CA, USA) was used for one-way ANOVA and Bonferroni’s post-hoc test where appropriate. Concentration-relaxation curves were analyzed by two-way ANOVA followed by Bonferroni post-tests. pD_2_ is the negative logarithm of the dilator concentration needed to cause 50% the maximal relaxation as determined by non-linear regression curve fitting. *p* < 0.05 was considered significant.

## 4. Conclusions

The present study demonstrated that early administration with Rho kinase inhibitor, fasudil significantly improved diabetes-related endothelial dysfunction in intrarenal arteries, without affecting metabolic indices in the STZ-induced diabetic rats. In addition, the chronic treatment with fasudil ameliorated mesangial matrix expansion in the kidney cortex from diabetic rats. Mechanically, the alleviated endothelial function and development of nephropathy may due to down-regulation of oxidative stress in the kidney. These findings provide new evidence to support Rho kinase inhibitors as potential therapeutic value for the treatment of diabetic nephropathy.

## Figures and Tables

**Table 1:** Primers used for real-time PCR.

Primer	Forward oligonucleotides	Reverse oligonucleotides
*P22phox*	CCTCCACTTACTGCTGTCCG	GTAGGTGGCTGCTTGATGGT
*P47phox*	GTCGGAGAAGGTGGTCTACAG	TCTTCACCTGGCTGTCATTGG
*Gp91phox*	CTGCCAGTGTGTCGGAATCT	TGTGAATGGCCGTGTGAAGT
*Nox3*	GCTGGGATGAATACCAGGCA	GCTGCTGCTAGGGTGATTGT
*Nox4*	CTGACAGGTGTCTGCATGGT	ACTTCAACAAGCCACCCGAA
*β-actin*	AGATCAAGATCATTGCTCCTCCT	ACGCAGCTCAGTAACAGTCC
